# Multiplex Immunofluorescence Captures Progressive Immune Exhaustion with Advancing Penile Squamous Cell Cancer Stage

**DOI:** 10.3390/cancers16020303

**Published:** 2024-01-11

**Authors:** Filip Ionescu, Jonathan Nguyen, Carlos Moran Segura, Mahati Paravathaneni, G. Daniel Grass, Peter Johnstone, Niki M. Zacharias, Curtis A. Pettaway, Xin Lu, Youngchul Kim, Junmin Whiting, Jasreman Dhillon, Steven A. Eschrich, Juskaran Chadha, Keerthi Gullapalli, Gabriel Roman Souza, Hiroko Miyagi, Brandon J. Manley, Philippe E. Spiess, Jad Chahoud

**Affiliations:** 1 Genitourinary Oncology Department, H. Lee Moffitt Cancer Center, Tampa, FL 33612, USA; filip.ionescu@moffitt.org (F.I.); mahati.paravathaneni@moffitt.org (M.P.); juskaran.chadha@moffitt.org (J.C.); philippe.spiess@moffitt.org (P.E.S.); 2 Pathology Department, H. Lee Moffitt Cancer Center, Tampa, FL 33612, USA; 3 Radiation Oncology Department, H. Lee Moffitt Cancer Center, Tampa, FL 33612, USA; 4 Department of Urology, University of Texas MD Anderson Cancer Center, Houston, TX 77030, USA; 5 Department of Biological Sciences, University of Notre Dame, Norte Dame, IN 46556, USA; xlu@nd.edu; 6 Biostatistics and Bioinformatics Department, H. Lee Moffitt Cancer Center, Tampa, FL 33612, USA

**Keywords:** penile squamous cell carcinoma, multiplex immunofluorescence, immune microenvironment

## Abstract

**Simple Summary:**

Penile cancer is a rare and aggressive disease. Current treatment options when the cancer is locally advanced are suboptimal and potentially mutilating. Insight into the immune dysregulation necessary for the emergence of penile cancer could suggest innovative ways to manipulate the immune system which have already demonstrated efficacy in other, more common malignancies. In this paper, we use multiplex immunofluorescence, a novel technology, to investigate the immune microenvironment of penile cancer for the first time. We describe a pattern of immune exhaustion as cancer becomes more advanced and identify tumor-associated macrophages as a potential key player in regulating this process.

**Abstract:**

Penile squamous cell carcinoma (PSCC) is a rare and deadly malignancy. Therapeutic advances have been stifled by a poor understanding of disease biology. Specifically, the immune microenvironment is an underexplored component in PSCC and the activity of immune checkpoint inhibitors observed in a subset of patients suggests immune escape may play an important role in tumorigenesis. Herein, we explored for the first time the immune microenvironment of 57 men with PSCC and how it varies with the presence of human papillomavirus (HPV) infection and across tumor stages using multiplex immunofluorescence of key immune cell markers. We observed an increase in the density of immune effector cells in node-negative tumors and a progressive rise in inhibitory immune players such as type 2 macrophages and upregulation of the PD-L1 checkpoint in men with N1 and N2-3 disease. There were no differences in immune cell densities with HPV status.

## 1. Introduction

Penile cancer is a rare and potentially deadly malignancy. Over 2000 new cases will be diagnosed in the United States 2023, and it is expected that 470 American men will die from the disease [1]. With incidence estimates of greater than 50,000 cases worldwide in 2023 following a steady increase in underdeveloped countries in Africa and South America, penile cancer is emerging as a global problem. Over 95% of penile malignancies are classified histologically as penile squamous cell carcinomas (PSCC), the treatment of which often results in devastating disfigurement, and only half of all patients survive beyond 5 years [2]. Risk factors associated with PSCC mainly include phimosis, tobacco use, and infection with human papillomavirus (HPV) [3,4]. HPV is involved in about half of PSCC cases. Its presence has direct impact on the genomic pathways involved in oncogenesis, but the impact of HPV infection on the immune environment of PSCC is incompletely characterized [3,5,6,7].

While localized PSCC can be treated with organ-sparing surgical techniques with variable impact on sexual function, standard treatment regimens for locoregionally advanced PSCC consist of multimodal therapies combining neoadjuvant chemotherapy (NAC) or chemoradiation (NACRT) regimens followed by curative intent surgery [8,9,10,11]. Unfortunately, more than 80% of these patients will experience disease relapse or have primary refractory disease to front-line chemotherapy, representing a sizeable subgroup with short survival of less than 6 months and very few effective salvage treatments [9,12]. The latter represents a clinical scenario of great unmet need, owing primarily to incomplete understanding of PSCC biology.

Immune checkpoint inhibitors have improved clinical outcomes in many solid tumors, but clinical activity in PSCC has been limited to only a small subset of patients [13,14,15]. The tumor immune microenvironment (TME) is a complex ecosystem that plays a critical role in cancer progression and response to immune therapies. In the case of PSCC, the immune cell changes that occur with advancing disease stage and in response to systemic therapies have not been well studied [16,17,18]. Characterization of the composition and cellular states of tumor-infiltrating immune cells has the potential to predict response to approved immune therapies and to potentially identify new targeting strategies for PSCC immunotherapy [19].

The current study used multiplex immunofluorescence (mIF) to describe the immune cellular composition of PSCC and to examine how it varies with disease stage, clinicopathologic features, and viral profiles.

## 2. Materials and Methods

A tissue microarray (TMA) was constructed for 57 cases of invasive PSCC, as previously described [20]. High-risk HPV was detected by in situ hybridization (ISH) [21]. mIF analysis was performed using formalin-fixed and paraffin-embedded (FFPE) tissue samples, which were immunostained for 10 immune markers, CD20, CD3, CD4, CD8, CD45RO, CD68, CD206, CD163, NKp46, and FOXP3, using the AKOAYA Biosciences OPAL TM 7-Color Automation IHC kit (Waltham, MA, USA) on the BOND RX autostainer (Leica Biosystems, Vista, CA, USA) [22]. Several prior investigations have reported on the ability of combinations of these markers to predict response to immune checkpoint inhibition on various tumor types [23,24,25]. The OPAL 7-color kit uses tyramide signal amplification (TSA) conjugated to individual fluorophores to detect various targets within the multiplex assay. Sections were baked at 65 °C for one hour, then transferred to the BOND RX (Leica Biosystems). All subsequent steps (ex., deparaffinization, antigen retrieval) were performed using an automated OPAL IHC procedure (AKOYA). Autofluorescence slides (negative control) were included, which use primary and secondary antibodies omitting the OPAL fluorescence and DAPI. All slides were imaged with a Vectra^®^3 Automated Quantitative Pathology Imaging System. Multi-layer TIFF images were exported from InForm (AKOYA) and loaded into HALO Image Analysis Platform (Indica Labs, Albuquerque, NM, USA) for quantitative image analysis. After setting a positive fluorescent threshold for each staining marker, the entire image set was analyzed with the created algorithm. The generated data included positive cell counts for each fluorescent marker in the cytoplasm or nucleus, and the percentage of cells positive for the marker. A classifier was trained to identify areas of tumor, stroma, or non-tissue regions using random forest supervised machine learning. The data were sorted by classification to include entire tissue, stroma, or tumor regions. PD-L1 expression was assessed at the TMA slide rather than the cellular level using immunohistochemistry, and quantified using Quickscore [26]. Finally, we created chord plots to visualize cell phenotypes’ interactions based on the co-expression of markers using the markers from each mIF panel [27].

Numerical variables had non-normal distributions and, consequently, are reported as median values (interquartile range [IQR]); Kruskal–Wallis tests were applied for comparisons. All *p*-values were two-sided and *p <* 0.05 was considered to indicate statistical significance. Statistical analysis was performed using JMP (software version 17.0.0) and R statistical software (version 4.2.2). Figures were created using GraphPad Prism (software version 9.5.1).

## 3. Results

We analyzed expression of 10 markers (CD20, CD3, CD4, CD8, CD45RO, CD68, CD206, CD163, NKp46, FOXP3) across two mIF panels within a single TMA for 57 men with PSCC. As noted, PD-L1 status was assessed on the same TMA slide using IHC. The baseline patient characteristics are presented in Table 1. The median age was 60 years, with a range from 31 to 92 years. Forty percent of tumors were positive for HPV. Most patients had involved lymph nodes on pathologic examination (31/57 [54%]) and, among those with uninvolved lymph nodes, 21/26 had intermediate or high-risk disease (T1 and grade 3 or ≥T2). Nearly one third of patients (17/57 [30%]) received adjuvant therapy: 6 were treated with cisplatin-based combination chemotherapy alone, 3 with radiotherapy alone, and 8 with chemoradiotherapy.

**Figure 1:**
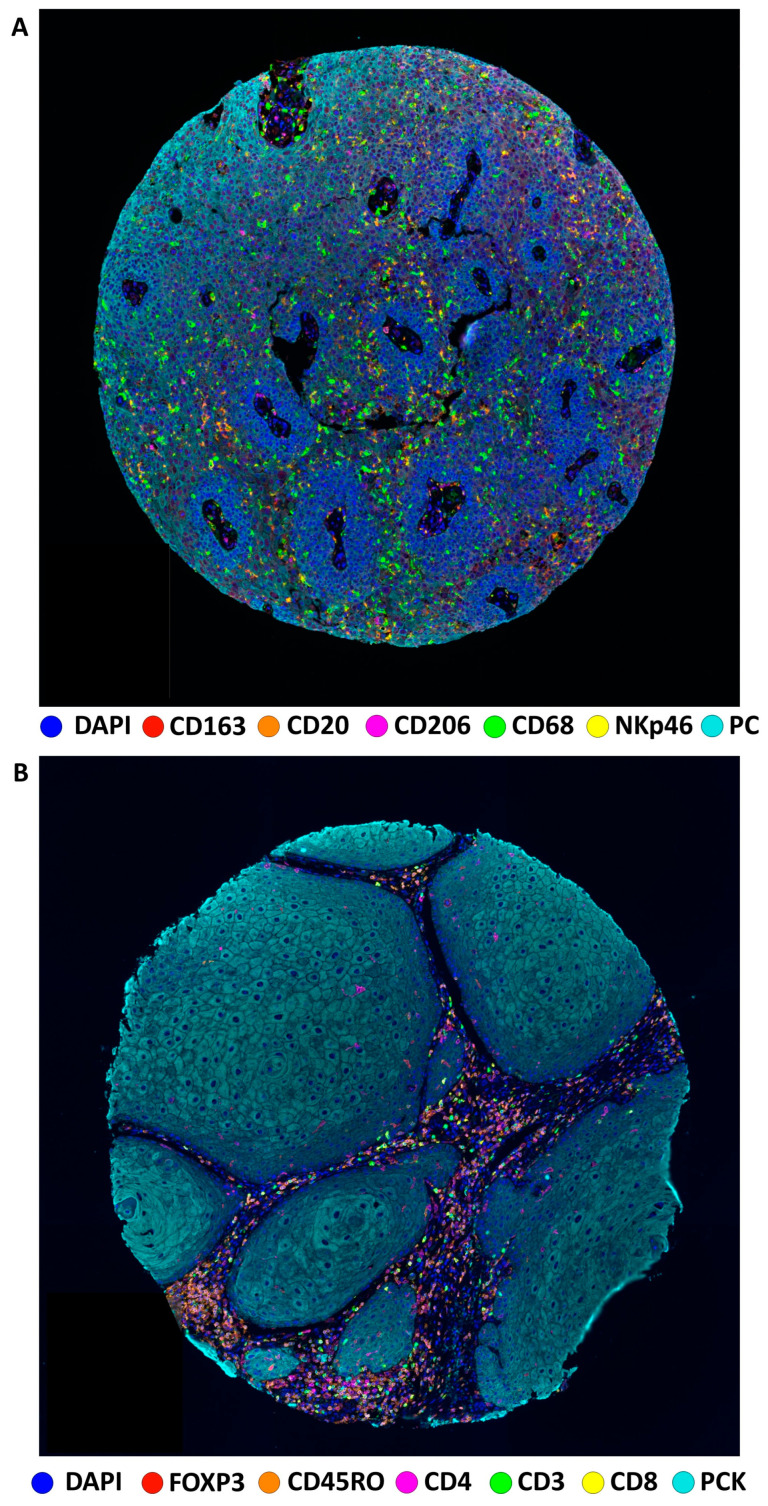
Representative tissue microarray micrographs of multiplex immunofluorescence panel 1 (**A**) and panel 2 (**B**).

**Figure 2:**
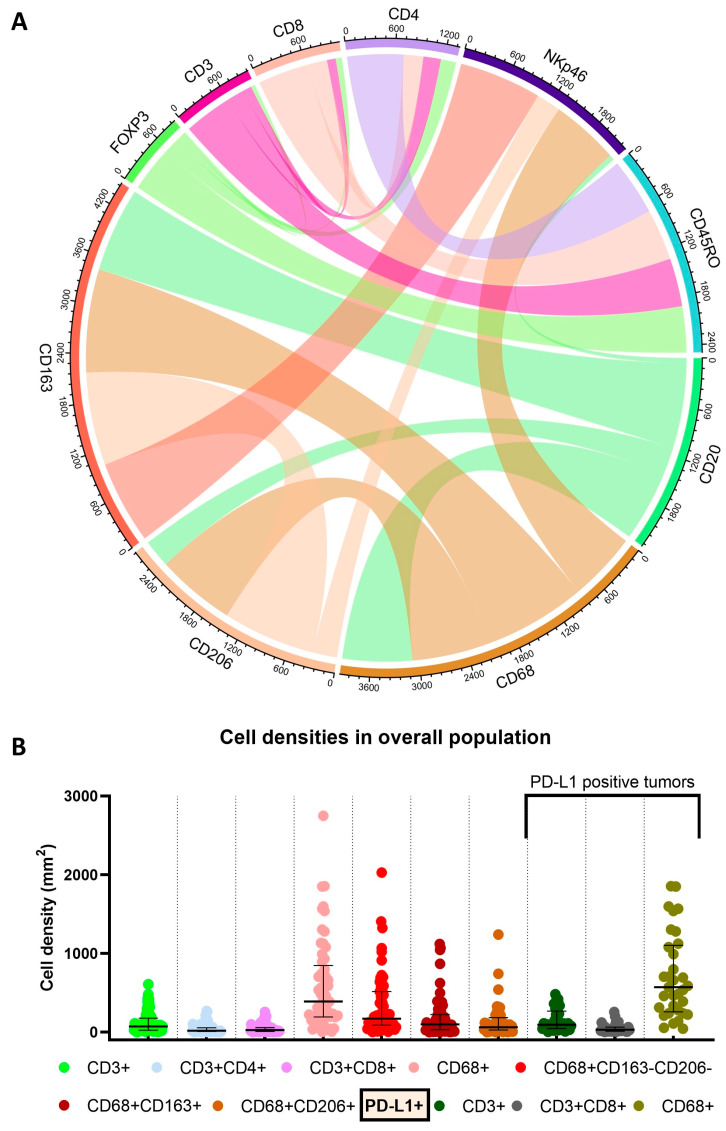
Chord plot of immune markers (**A**) and cell densities, as determined by multiplex immunofluorescence in all penile squamous cell cancer tumors and in PD-L1-positive tumors (**B**).

Figure 1 shows representative examples of multispectral images from PSCC tissue microarray specimens for the two mIF panels (Figure 1A,B), while a chord diagram of the markers (Figure 2A) and the distribution of cell densities in the overall population (Figure 2B) are depicted in Figure 2.

**Figure 3:**
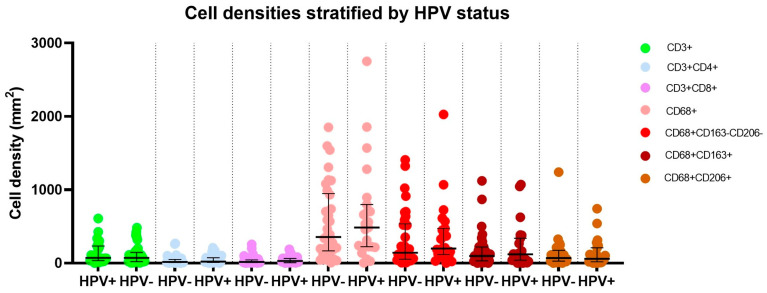
Cell densities as determined by multiplex immunofluorescence in HPV-negative and HPV-positive penile squamous cell cancer patients.

Densities did not differ significantly for any cell type of interest between HPV-positive and HPV-negative patients (Figure 3). Of note, however, HPV-positive tumors had nearly double the median density of CD3+CD8 + or cytotoxic T-cell phenotype in all tumors (28.2 vs. 15 cells/mm^2^; *P* = 0.34) and in PD-L1-positive or immunologically exhausted tumors (39.2 vs. 21.9 cells/mm^2^; *P* = 0.21). HPV-positive patients had similar densities of CD68+ cells (total macrophages), CD68+CD163+, and CD68+CD206+ (type 2 macrophages).

**Figure 4:**
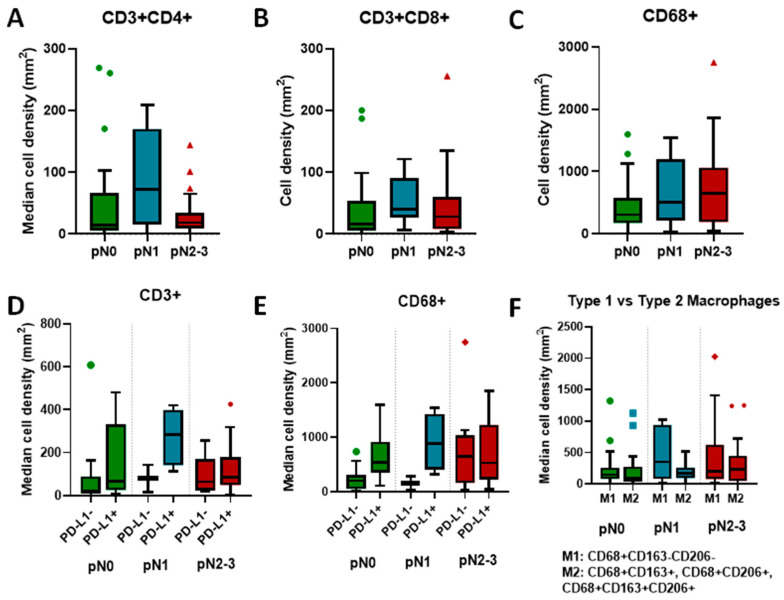
Median cell densities across stages for CD3+CD4+ helper T cells (**A**), CD3+CD8+ cytotoxic T cells (**B**), and CD68+ macrophages (**C**). Panels (**D**,**E**) show the density of all CD3+ T cells (**D**) and CD68+ macrophages (**E**) stratified by both stage and PD-L1 status of the tumor. Panel (**F**) compares densities of CD68+CD163−CD206− macrophages (type 1) and CD68+, and either CD206+ or CD163+ or both macrophages (type 2) across clinical stages, stratified by PD-L1 status.

We investigated cell densities by clinical stage and found an increase in the density of all T-cells (CD3+), as well as helper (CD3+CD4+, Figure 4A) and cytotoxic (CD3+CD8+, Figure 4B) T-cell subsets going from localized (pN0: CD3 44.23, CD4+ 14.77, CD8+ 16.15 cells/mm^2^) to regional (pN1: CD3+ 188.36, CD4+ 71.69, CD8+ 40.22 cells/mm^2^) disease. In more extensive regional disease (pN2), however, the density of T-cells decreased (CD4+ 18.45 and CD8+ 28.22 cells/mm^2^). By contrast, the median density of total macrophages increased with stage (pN0: 306.38; pN1: 502.47; pN2-3: 648.27 cells/mm^2^; Figure 4C). Across stages, PD-L1-positive tumors had greater densities of T-cells (CD3+) and macrophages (CD68+) with a larger density gradient in the N1 stage and a smaller gradient in the N2-3 stage (Figure 4D,E). Notably, the macrophage phenotype switched across stages (Figure 4F). Whereas in localized disease (pN0) there were more activated M1 anti-tumor macrophages (CD68+CD163–CD206–), cell number continued to increase in early locoregional disease (pN1); the median density of cancer-promoting M2 macrophages (CD68+CD163+, CD68+CD206+, CD68+CD163+CD206+) increased steadily across stages and became the dominant macrophage type in the pN2-3 setting. The median densities for markers of interest are presented in Table 2.

## 4. Discussion

In this study, we performed mIF analysis to characterize the immune microenvironment of PSCC to describe the interplay between different immune effector cells as it varies with the presence of HPV infection, and how it evolves across clinical stages. To our knowledge, this is the first such undertaking of its kind in this rare and aggressive malignancy.

In evaluating the immune environment according to disease spread from localized (pN0) to early locoregional (pN1) and more advanced locoregional (pN2-3) disease, we observed substantial differences in the densities of different immune actors. In the transition from pN0 to pN1, there was a nearly 50-fold increase in the median density of all lymphocytes, including in individual subsets of T helper and T effector cells, consistent with the generation of an immune response. In the N1 stage, however, the percentage of PD-L1 positive tumors also increased, indicative of the emergence of immune tolerance or exhaustion. Subsequently, in the pN2-3 stage, we observed a decline in lymphocyte levels close to the pre-immune response pN0 levels. Concomitantly, we recorded notable changes in the myeloid component of the microenvironment. The median density of macrophages increased across stages, more than doubling from the pN0 (306.38 cells/mm^2^) to the pN2-3 stage (648.28 cells/mm^2^). Similar to the lymphoid counterpart, the median density macrophages in PD-L1-positive tumors increased in the pN1 stage and accompanied a switch from anti-tumor M1 macrophages to pro-tumor M2 macrophages, the latter of which increased steadily across stages and outnumbered the M1 population in the pN2-3 stage.

This analysis is the first to suggest the emergence of immune exhaustion in PSCC with advancing stages, and it suggests an important role for myeloid cells in creating an immunotolerant, pro-tumor environment. M2 macrophages have numerous and diverse pro-tumor functions which act at different phases of carcinogenesis [28,29,30]. Beyond secretion of growth factors for cancer stem cells and proteases, which increase the laxity of the extracellular matrix and promote metastatic spread, M2 macrophages also release mediators such as IL-10, TGFβ, prostaglandins, and indoleamine 2,3-dioxygenase, which promote the expansion of regulatory T cells and metabolically starve effector T-cells. In addition, they also exhibit a high expression of immunosuppressive immune checkpoint molecules (PD-L1, PD-L2, B7-H4), directly causing T cell exhaustion [28,31]. However, the modest activity of PD-L1 inhibition in the PERICLES trial suggests that immune escape in PSCC may occur through other mechanisms than the PD-1 and CTLA-4 pathways [15,32,33]. Our results provide insight into one such potential alternative pathway by suggesting a role for the macrophage phenotype switching as it develops over advancing clinical stages in PSCC. This observation opens up exciting avenues for further investigations in PSCC, focused on further exploring the biology of macrophage–lymphocyte interactions and the benefit of macrophage-targeting strategies such as pharmacologically targeting the macrophage metabolism or surface checkpoints, which have shown promising anti-tumor activity in early studies [28,34,35,36].

mIF is increasingly employed to study the tumor immune microenvironment. Investigations of squamous cell carcinomas of other sites (head and neck, cervix, and lung) have both advanced our understanding of how immune evasion occurs and, in some analyses, have shown their potential in predicting response to immune therapies and aiding clinical decision making. In head and neck squamous cell carcinomas, mIF revealed that smokers had significantly lower numbers of CD8+ cytotoxic T cells and PD-L1+ cells in the tumor microenvironment compared with never-smokers and former smokers, likely secondary to suppression of interferon pathways [37]. Two independent studies have shown that higher tumor densities of CD8+ T cells are associated with better prognosis [38,39]. Interestingly, mIF exploration of oropharyngeal squamous cell carcinoma revealed that HPV-positive lesions were more heavily infiltrated with CD8+ T-cells and had higher PD-L1 expression, as compared to HPV-negative counterparts [40]. Moreover, CD8+ T cells were in closer proximity to tumor cells and CD163+ macrophages, and PD-L1+ tumor cells and macrophages were closer to PD-1+ cytotoxic T lymphocytes in HPV-positive primary tumors. In our cohort, we found nearly double the density of CD8+ T-cells in HPV-positive PSCC lesions (observed in both PD-L1-positive and PD-L1-negative tumors), yet the difference did not prove statistically significant and, indeed, the overall composition of immune cells was similar regardless of HPV status in PSCC. This finding suggests that, while HPV may play a role in carcinogenesis, the process of immune escape may be analogous to that of HPV-negative cases. A similar progression across clinical stages as that observed in PSCC, with increasing density of CD68+ and decreasing CD8+ cells from localized tumors to nodal metastases, was reported in cervical squamous cell carcinoma [41]. The authors propose an mIF-based nomogram to identify cervical cancer patients at risk of nodal metastases and note the very high discriminatory ability of such an approach. Insights from mIF performed in lung cancer suggest that higher densities of CD8+ T-cells and PD-L1+ cells, especially when co-expressed, are significantly associated with significantly longer PFS and OS when patients are treated with immunochemotherapy [42,43]. Whether a similar relationship can be demonstrated in PSCC remains uncertain, but these observations could inform future efforts to better select PSCC patients for such therapies, and improve the modest response rates observed in unselected populations [15]. Finally, one important observation pertaining to mechanisms of immune escape stems from mIF analyses of lung cancer specimens, which show significantly greater infiltration by myeloid-derived immunosuppressive cells in squamous as opposed to adenocarcinoma histology [44]. As outlined above, the specific targeting of this axis of the immune system could prove important to improving the efficacy of immune therapies in squamous histologies.

The key strengths of our study are the application of mIF, a novel quantitative technique able to simultaneously detect multiple markers on a single tissue section and provide a comprehensive description of cell composition, in a relatively large number of subjects with a rare malignancy. In absolute terms, however, our sample size is expectedly small, which likely limited our ability to definitively detect statistical differences between groups; future validation studies are needed. Important complementary investigations such as geospatial analysis and RNA sequencing, as well as the impact of these immunophenotypic and transcriptomic correlative data on clinical outcomes, are underway and will be reported in subsequent publications.

## 5. Conclusions

In this study, we investigated the immune microenvironment of PSCC for the first time using a novel mIF technique. We describe a stepwise process of immune exhaustion characterized by initial infiltration by effector immune cells in localized tumors and progressive accumulation of immune inhibitory players (chiefly tumor-associated macrophages), a decline in effector cell densities, and a rise in inhibitory checkpoint signal expression with a greater degree of nodal involvement. We found no differences in the densities of infiltrating immune cells with HPV status, suggesting that the impact of viral infection does not extend to the immune escape process occurring following tumorigenesis. Our subsequent efforts will focus on geospatial analysis and determining the transcriptomic changes that occur in the tumor to allow for immune escape in PSCC. These findings support continued investigations into treatment strategies for immune manipulation in PSCC.

## Figures and Tables

**Table 1:** Baseline characteristics of the overall population (N = 57).

Median age in years at surgery (IQR, range)	59.9 (53.1–73.4, 30.5–92.0)
White race	44 (77%)
HPV positive	23 (40%)
P16 positive	24 (42%)
Primary surgery	
Partial penectomy ± scrotectomy	40 (70%)
Total penectomy	12 (21%)
Wide local excision	4 (7%)
Circumcision	1 (2%)
Grade	
1	38 (67%)
2	16 (28%)
3	3 (5%)
Lymphovascular invasion present	32 (56%)
pT stage	
1	17 (30%)
2	16 (28%)
3	22 (39%)
4	2 (3%)
pN stage	
0	26 (46%)
1	6 (10%)
2	20 (35%)
3	5 (9%)

**Table 2:** Cell marker densities across clinical stage.

Median Marker Density/mm^2^ (Range)	pN0	pN1	pN2-3	*p*-Value
CD3+	44.2	188.4	77.9	0.15
CD3+CD4+	14.8	71.7	18.5	0.29
CD3+CD8+	16.2	40.2	28.2	0.19
CD68+	306.4	502.5	648.3	0.28

## Data Availability

Deidentified data are available upon request.
